# Factors associated with severe infection in rheumatoid arthritis patients: lessons learned from the COVID-19 pandemic

**DOI:** 10.1007/s15010-024-02187-z

**Published:** 2024-02-21

**Authors:** Aya Embaby, Lobna A. Maged, Hoda M. Abdel-Hamid, Khaled T. El Hadidi

**Affiliations:** 1 Rheumatology Department, Kasr Al Ainy Faculty of Medicine, Cairo University, Cairo, Egypt; 2 Chest Department, Kasr Al Ainy Faculty of Medicine, Cairo University, Cairo, Egypt

**Keywords:** Coronavirus disease 2019 (COVID-19), Rheumatoid arthritis (RA), Severe infection

## Abstract

**Purpose:**

This aimed to identify the factors associated with severe/critical coronavirus disease 2019 (COVID-19) infection in rheumatoid arthritis (RA) patients.

**Methods:**

Two-hundred RA patients diagnosed according to the American College of Rheumatology/ European League Against Rheumatism (ACR/EULAR) classification criteria with proven COVID-19 infection were recruited and categorized according to the world health organization (WHO) COVID-19 severity grading into 2 groups: patients with mild/moderate COVID-19 (*n* = 164) and patients with severe/critical COVID-19 (*n* = 36). Comparison between both groups was done to identify the risk factors associated with severe/critical infection. Incidence of RA disease activity flare defined as increase in clinical disease activity index (CDAI) more than 10 points following infection was calculated.

**Results:**

Multivariate analysis identified history of previous serious infection, age > 60 years, and diabetes as factors positively associated, whereas COVID-19 vaccination was negatively associated with severe/critical infection. Following COVID-19 infection, the number of patients with severe/critical COVID-19 who had high RA disease activity and the incidence of flares was significantly higher in comparison to patients with mild/moderate COVID-19 (*P* < 0.001 and 0.003; respectively).

**Conclusion:**

Age > 60 years, diabetes, and history of previous serious infections are risk factors for severe/critical COVID-19, while vaccination has a protective role in RA patients. Infection particularly when severe is associated with risk of disease flare.

## Introduction

Serious infections (SIs) in patients with rheumatoid arthritis (RA) are a major concern with risk of increased overall mortality. The immunological dysfunction of the disease itself, concomitant illnesses, and/or immunosuppressive medicines may all contribute to the higher risk of SIs in RA [1, 2].

The coronavirus disease 2019 (COVID-19) pandemic, caused by severe acute respiratory syndrome coronavirus 2 (SARS-CoV-2), was one of the greatest threats to public health in the century [3]. The amount of research invested in COVID-19 was huge, and effective vaccines and potential therapeutic agents were discovered [4, 5]. Despite that, until January 2023 COVID-19 was still declared a “Public Health Emergency of International Concern (PHEIC)” [6]. The end of the global emergency was declared in May 2023, but the health community was instructed to remain vigilant [7]. Typically, the virus causes a mild to moderate respiratory illness. However, it occasionally leads to severe alveolar disease that can substantially lead to respiratory failure [8].

The similarities in the cytokine profile, patterns of immune activation, and some common therapeutic strategies suggest an intricate relationship between COVID-19 and RA [9, 10]. Evidence revealed an increased risk of COVID-19 generally and serious outcomes specifically in RA patients [11]. On the other hand, infections are among the environmental factors that can trigger RA [12]. There are numerous cases reported concerning the development of RA following COVID-19 infection [13]. In patients with pre-existing RA, infection can induce flares [14].

The pandemic should not pass without lessons. The data available regarding the co-existence of both diseases can be further invested to elucidate the bidirectional relationship between infections and RA. The current study aimed to identify the factors associated with severe COVID-19 infection in RA patients. The risk of flares in patients following infection was considered a secondary objective.

## Patients and methods

This study is a case control study in which RA patients, diagnosed according to 2010 American College of Rheumatology/ European League Against Rheumatism (ACR/EULAR) classification criteria [15], infected with COVID-19 were recruited. Infection was proven by either positive COVID-19 polymerase chain reaction (PCR), SARS-CoV-2 immunoglobulins IgM/IgG or typical computed tomography (CT) chest findings with classic symptoms and signs [16]. Patients were recruited from Rheumatology and Rehabilitation Department, Faculty of medicine, Cairo University hospitals and private rheumatology centers. After analysis of records and personally contacting 1864 patients; 200 patients had a proven COVID-19 infection. They were infected from the start of pandemic in 2019 till 30 June 2022. Patients were informed about the objective of the study and oral consent was obtained for the use of medical data.

Grading of severity of COVID-19 infection was done according to the WHO disease severity grading [16]. Patients who had severe/critical COVID-19 were compared to those with mild/moderate COVID-19 to determine factors associated with severe infection. Comparison was done in terms of general characteristics including age, gender, comorbidities, history of previous serious infections defined as infections that required hospitalization and/or intravenous antibiotics, COVID-19 vaccination prior to infection, as well as RA-related disease factors including disease duration, seropositivity, presence of extra-articular manifestation, disease activity grading prior to infection measured using the Clinical Disease Activity index (CDAI) [17, 18], and medications used to treat RA. Factors that turned out to be significantly different between both groups were subjected to multivariate analysis.

Disease activity grading using CDAI within 6 months prior to COVID-19 infection and within 6 months following infection was obtained from patients’ records and a comparison between both readings was used to calculate the incidence of RA flare. Asai and colleagues defined flare as a CDAI score > 10 [19]. It is also the cut-off for moderate disease activity in the originator score [18]. More than 50% of our cohort had moderate/ high disease activity at baseline and hence increase of CDAI score > 10 points rather than the absolute value was considered flare.

### Statistical analysis

Data were coded and entered using the statistical package for the Social Sciences (SPSS) version 26 (IBM Corp., Armonk, NY, USA). Data were summarized using mean, standard deviation, median, minimum, maximum and interquartile range in quantitative data and using frequency (count), and relative frequency (percentage) for categorical data. Comparisons between quantitative variables were done using the non-parametric Kruskal–Wallis and Mann–Whitney tests. For comparing categorical data, Chi square (*χ*^2^) test was performed. Exact test was used instead when the expected frequency is less than 5. Multivariate logistic regressions were done to determine the risk factors associated with the occurrence of severe, critical COVID-19 infection. *P* values less than 0.05 were considered statistically significant.

## Results

The demographic data, baseline clinical characteristics, and treatment received by the studied patients are shown in Table 1. The majority of patients were females (93.5%) and their mean age was 48.92 ± 12.1 years. The most common symptoms of COVID-19 infection were myalgia and bone aches, followed by fatigue and sore throat in 74%, 57.5%, and 55.5% of patients, respectively. Most patients had mild COVID-19, while 36 (18%) patients had severe/critical COVID-19 (Table 2).

**Table 1:** Demographic data and baseline characteristics of patients

Variable	
Age, Mean ± SD	48.92 ± 12.1
Sex, *N* (%)	
Male	13 (6.5)
Female	187 (93.5)
Residence, *N* (%)	
Rural	44 (26.8)
Urban	120 (73.2)
BMI, Mean ± SD	31.01 ± 5.91
BMI categories, *N* (%)	
Underweight	4 (2)
Normal	23 (11.5)
Overweight	66 (33)
Obese	107 (53.5)
Smoking, *N* (%)	5 (3)
Comorbidities, *N* (%)	
Hypertension	35 (17.5)
Diabetes	16 (8)
Bronchial asthma	22 (11)
Hypothyroidism	13 (6.5)
Hypertension	25 (15.2)
Cardiac disease	6 (3)
History of previous serious infection, *N* (%)^a^	42 (21)
COVID-19 vaccine prior to infection, *N* (%)	41 (20.5)
RA-related factors	
Disease duration, Median (IQ range)	7 (4:11)
Seropositivity (RF/Anti-CCP), *N* (%)	116 (58)
RF, *N* (%)	109 (54.5)
Anti-CCP, *N* (%)	45 (22.5)
Extra-articular manifestation, *N* (%)	
2ry Sjogren syndrome	25 (12.5)
Interstitial lung disease	4 (2)
Subcutaneous nodules	8 (4)
RA disease activity^b^	
CDAI before COVID-19, Median (IQ range)	15 (6:25)
RA disease activity grading^b^, *N* (%)	
Remission	22 (11)
Low disease activity	60 (30)
Moderate disease activity	60 (30)
High disease activity	61 (30.5)
Medications before infection, *N* (%)	
Prednisolone ≤ 5 mg	134 (67)
Prednisolone > 5 mg	12 (6)
Hydroxychloroquine	105 (52.5)
Methotrexate	96 (48)
Leflunomide	114 (57)
Sulfasalazine	3 (1.5)
Combination conventional DMARDs	113 (56.5)
Anti-TNF^c^	27 (13.5)
Etanercept^c^	23 (11.5)
Golimumab^c^	4 (2)
Rituximab^c^	2 (1)
JAK inhibitor^c^	3 (1.5)
Combination of biologic/targeted DMARD with conventional DMARD^c^	32 (16)

*SD* Standard deviation, *IQ* Interquartile, *BMI* Body mass index, *COVID-19* Corona virus disease 2019, *RA* Rheumatoid arthritis, *RF* Rheumatoid factor, *Anti-CCP* Anti-cyclic citrullinated peptide, *CDAI* Clinical Disease Activity Index, *TNF* Tumor necrosis factor, *JAK* Janus kinase, *DMARD* Disease-modifying anti-rheumatic drug

^a^Previous serious infection is infection that required hospitalization and/or IV antibiotics

^b^RA disease activity was categorized according to the CDAI within 6 months before COVID-19 infection

^c^All patients on a biologic/targeted DMARD received it in combination with a conventional DMARD.

**Table 2:** Clinical manifestations, severity, and diagnostic tests used to prove COVID-19 infection

COVID-19 manifestations	*N*	%
Clinical presentation		
Fever	110	55
Fatigue	115	57.5
Sore throat	111	55.5
Nasal congestion	68	34
Anosmia	45	22.5
Ageusia	39	19.5
Cough	110	55
Dyspnea	110	55
Myalgia, Bony aches	148	74
Gastrointestinal symptoms	24	12.1
Sepsis, septic shock	2	1
Grading of severity^a^		
Mild	89	44.5
Moderate	75	37.5
Severe	32	16
Critical	4	2
Mortality	3	1.5
Diagnostic tools to prove COVID-19 infection		
PCR	56	28
COVID-19 immunoglobulin test	54	27
CT chest with typical symptoms and signs	100	50

*COVID-19* Corona virus disease 2019, *PCR* Polymerase chain reaction, *CT* Computed tomography

^a^Severity of COVID-19 was classified according to the WHO classification [who.in]

A comparison between patients with mild/moderate COVID-19 (*n* = 164) and those with severe/critical COVID-19 (*n* = 36) was done and is demonstrated in Table 3. Factors that were significantly different between both groups in the univariate analysis were subjected to multivariate analysis as demonstrated in Table 4. History of previous serious infection, age > 60 years, and diabetes had a positive association, whereas COVID-19 vaccination was negatively associated with severe/critical COVID-19 infection. Patients with severe/critical COVID-19 had a significantly higher CDAI prior to infection when compared with those with mild/moderate COVID-19. However, after adjustment of confounding factors CDAI prior to COVID-19 was no longer significant.

**Table 3:** Comparison of demographic data and clinical manifestations and treatment in patients with different grades of severity of COVID-19

	Mild, moderate COVID-19 *N* = 164	Severe, critical COVID-19 *N* = 36	*P* value
Age (years), Mean ± SD	47.37 ± 11.60	56.14 ± 11.71	< 0.001
Age groups (years), *N* (%)			
18–30	12 (7.3)	0 (0)	0.129
> 30–50	86 (52.4)	10 (27.8)	0.007
> 50–60	44 (26.8)	10 (27.8)	0.908
> 60	22 (13.4)	16 (44.4)	< 0.001
Sex, *N* (%)			0.80
Male	11 (6.7)	2 (5.5)	
Female	153 (93.3)	34 (94.4)	
BMI, Mean ± SD	30.64 ± 5.71	33.10 ± 6.27	0.009
BMI categories, *N* (%)			
Underweight	4 (2.4)	0 (0)	1
Normal	22 (13.4)	1 (2.8)	0.085
Overweight	56 (34.1)	10 (27.8)	0.462
Obese	82 (50)	25 (69.4)	0.034
Comorbidities, *N* (%)			
Bronchial asthma	18 (11)	4 (11.1)	1
DM	9 (5.5)	7 (19.4)	0.012
Hypothyroidism	12 (7.4)	1 (2.8)	0.47
Hypertension	25 (15.2)	10 (27.8)	0.073
Cardiac disease	2 (1.2)	4 (11.1)	0.01
Treatment with ACE inhibitors, ARBs	2 (1.2)	2 (5.6)	0.149
History of previous serious infection^a^, *N* (%)	22 (13.4)	20 (55.6)	< 0.001
COVID-19 vaccine prior to infection, *N* (%)	40 (24.4)	1 (2.8)	0.004
RA-related factors			
Disease duration, Median (IQ range)	6.5 (4:11)	8 (4:12)	0.186
Extra-articular manifestations, *N* (%)			
2ry Sjogren syndrome	20 (12.2)	5 (13.9)	1
ILD	2 (1.2)	2 (5.6)	0.149
SC nodules	5 (3)	3 (8.3)	0.157
Seropositivity (RF/Anti-CCP), *N* (%)	92 (56.1)	24 (66.7)	0.244
CDAI before COVID-19, Median (IQ range)	13 (6:23.75)	26 (17:38)	< 0.001
RA disease activity grading^b^, *N* (%)			
Remission and low disease activity	64	6	0.011
Moderate disease activity	45 (27.4)	12 (33.3)	0.478
High disease activity	45 (27.4)	16 (44.4)	0.045
Prednisolone dose before infection, Mean ± SD	2.29 ± 1.9	3.54 ± 2.66	< 0.001
Medications used before infection, *N* (%)			
Prednisolone ≤ 5 mg	112 (68.3)	22 (61.1)	0.407
Prednisolone > 5 mg	4 (2.4)	8 (22.2)	< 0.001
Hydroxychloroquine	89 (54.3)	16 (44.4)	0.285
Methotrexate	82 (50)	14 (38.9)	0.227
Leflunomide	88 (53.7)	26 (72.2)	0.042
Sulfasalazine	3 (1.8)	0 (0)	1
Rituximab^c^	0 (0)	2(5.6)	0.032
Etanercept^c^	19 (11.6)	4 (11.1)	1
Golimumab^c^	4 (2.4)	0 (0)	1
JAK inhibitor^c^	3 (1.8)	0 (0)	1
Combination conventional DMARDs	92	22	0.582
Combination biologic/targeted, conventional DMARDs^c^	26	6	0.90

*SD* Standard deviation, *IQ* Interquartile, *BMI* Body mass index, *COVID-19* Corona virus disease 2019, *RA* Rheumatoid arthritis, *RF* Rheumatoid factor, *Anti-CCP* Anti-cyclic citrullinated peptide, *CDAI* Clinical Disease Activity Index, *TNF* Tumor necrosis factor, *JAK* Janus kinase, *DMARD* Disease-modifying anti-rheumatic drug

^a^Previous serious infection is infection that required hospitalization and/or IV antibiotics

^b^RA disease activity was categorized according to the CDAI within 6 months before COVID-19 infection

^c^All patients on a biologic/targeted DMARD received it in combination with a conventional DMARD

**Table 4:** Multivariate analysis of factors associated with COVID-19 infection severity

	*P* value	Odds Ratio (OR)	95% confidence interval (CI)
Age > 60 years	< 0.001	6.13	2.12–17.72
Obesity	0.120	2.21	0.81–5.99
Diabetes Mellitus	0.040	4.54	1.07–19.32
Cardiac disease	0.547	2.14	0.18–25.52
History of previous serious infection^a^	< 0.001	5.88	2.13–16.22
COVID-19 vaccine prior to infection	0.028	0.06	0.005–0.73
CDAI before COVID-19^b^	0.326	1.02	0.98–1.05
Prednisolone > 5 mg	0.137	3.39	0.68–16.92
Leflunomide	0.621	1.86	0.67–5.15

*COVID-19* Corona virus disease 2019, *CDAI* Clinical Disease Activity Index

^a^Previous serious infection is infection that required hospitalization and/or IV antibiotics

^b^CDAI as a measure of RA disease activity within 6 months before COVID-19 infection

Following COVID-19 infection, the number of patients with high disease activity and the incidence of flares in the severe/critical COVID-19 group was significantly higher in comparison to patients with mild/moderate COVID (*P* < 0.001 and 0.003; respectively) (Table 5).

**Table 5:** Rheumatoid arthritis disease activity grading following COVID-19 infection

	Mild, moderate COVID-19 *N* = 164	Severe, critical COVID-19 and mortality *N* = 36	*P* value
CDAI after COVID-19^a^, Median (IQ range)	14 (7:25)	26 (16.5:38)	< 0.001
Remission, *N* (%)	57 (34.8)	3 (9.1)	0.003
Low disease activity, *N* (%)	52 (31.7)	9 (27.3)	0.684
Moderate disease activity, *N* (%)	52 (31.7)	9 (27.3)	0.615
High disease activity, *N* (%)	45 (27.4)	21 (63.6)	< 0.001
Flare of activity after COVID-19^b^, *N* (%)	27 (16.5)	13 (39.4)	0.003

*COVID-19* Corona virus disease 2019, *CDAI* Clinical Disease Activity Index, *IQ* Interquartile

^a^CDAI was done within 6 months following COVID-19 infection

^b^Comparison of CDAI value within 6 months prior to and after COVID-19 infection was done and increase of CDAI score > 10 points was considered flare.

## Discussion

COVID-19 was one of the largest pandemics faced by humans in the modern age [20]. There was a great concern about vulnerable groups including those with autoimmune disease [21]. The risk of COVID-19 in RA being one of the most common autoimmune rheumatic diseases was extensively studied [11]. In a study including 33,886 people with RA in the US Veterans Affairs system, the risk of COVID-19 diagnosis was 25% higher than in 33,886 people without RA [22].

Whether RA is associated with an increased risk of COVID-19 infection is beyond the scale of the current study. The study, however, was conducted to determine the factors associated with severe infection. Several studies conducted on RA patients showed increased risk of hospitalization, poor outcome, and death when compared to the general population [22–24]. However, after adjustment for demographic data and comorbidities the risk declined [25] or was even lost [26] in some studies.

In the current study older age, particularly age > 60 years was among the strongest predictors of severe infection (OR 6.13, 95% CI 2.12–17.72). Among the studied comorbidities, diabetes was also identified as a strong predictor (OR 4.54, 95% CI 1.07–19.32). Both factors were associated with poor outcome in a substantial number of studies conducted on the general population. In a meta-analysis that included 109 articles and 20,296 participants, both increasing age (RR 1.45, 95% CI 1.23–1.71) and diabetes (RR 1.59, 95% CI 1.41–1.78) were associated with higher risk of mortality. Every ten-year increase in the mean age of patients was associated with a 7.6% increase in the mortality and 11.2% increase in disease severity [27]. In a retrospective study conducted on 2724 Egyptian COVID-19 patients, of whom 423 (15.52%) were critically ill, age > 60 years (OR 1.30, 95% CI 1.05–1.61, *P* = 0.014), diabetes (OR 1.62, 95% CI 1.26–2.08, *P* < 0.001) were among the predictors of critical illness [28]. In the COVID-19 Global Rheumatology Alliance (C19-GRA) registry which included 3,729 patients with rheumatic disease, the risk of death was associated with age (66–75 years: OR 3.00, 95% CI 2.13–4.22; > 75 years: 6.18, 4.47–8.53; both vs ≤ 65 years) [29].

In COVID-19, age seems to be the most important determinant of infection risk possibly reflecting age-related reduction in expression of angiotensin-converting enzyme 2 (ACE-2) receptor, a cellular receptor for SARS- CoV-2 binding [30] age-dependent difficulty in removing particles from small airways [31], excessive release of inflammatory mediators in elderly “inflammaging” [32], incompetent immune response, and high frequency of comorbidities in the elderly population [33]. Several mechanisms render diabetics more prone to infections, particularly COVID-19 infection including defective chemotaxis, macrophage, and phagocytic activity and reduced secretion of cytokines. Poor glycemic control facilitates viral replication and hypoglycemic drugs were implicated in facilitating the entry of SARS-CoV-2 into cells and increased expression of ACE-2 receptor [34].

COVID-19 vaccination offered protection against severe/critical infection in the current study (OR 0.06, 95% CI 0.005–0.73). Most patients with rheumatic disease generate antibody responses after vaccination, although lower antibody titers might be produced than in the wider population [35, 36]. It should be noted, however, that the current study included patients infected with different strains of COVID-19 that could have contributed to variable degrees of severity. SARS-CoV-2 strains that circulated when COVID-19 vaccines became available were associated with decreased mortality in comparison to preceding waves [37].

The effect of RA-related disease activity on the severity of infection was of particular concern in the current study. The potential effect of disease activity found in univariate analysis was lost after adjustment for confounders. The limited insurance coverage in our country results in frequent use of steroids as demonstrated in the current study. Mean prednisolone dose was significantly higher and the use of prednisolone > 5 mg was more frequent in patients with severe/critical COVID-19; the difference was also lost after adjustment for confounders. Patients with a more aggressive disease receive more aggressive immunosuppressants including steroids, and hence, confounding by indication has always been a challenge faced when this particular issue was addressed. Several studies found an association between higher disease activity levels and risk of infection including COVID-19 infection in RA patients [2, 29, 38–40]. Strangfeld and colleagues could not find a direct association between higher disease activity and infection risk; the effect, however, was indirect due to steroid use and decline in function [41].

History of previous infection was identified as another predictor of severe COVID-19 (OR 5.88, 95% CI, 2.13–16.22). In a previous study analyzing the risk of serious infections in patients with musculoskeletal disorders, history of prior serious infections was associated with increased risk of subsequent infection [2]. Concomitant bacterial infections were frequently observed in patients with COVID-19 [42] particularly in older patients and those with chronic medical diseases [43], suggesting common pathophysiological mechanisms.

The relationship between COVID-19 and RA is bidirectional, and hence, the effect of COVID-19 on RA disease activity was also investigated in the current study. The hyperstimulation and dysregulation of the immune system in severe COVID-19 was implicated in substantially higher risk of development of autoimmune diseases including RA according to a recent study [44, 45] and might hence result in disease flare in patients who already have these conditions. The current study showed evidence of higher disease activity levels, particularly in patients with severe/critical infection. The fear of utilization of RA treatment during the pandemic and interruption of treatment in patients who developed COVID-19 infection are additional contributing factors.

The current study has the strength of being one of the few studies of its type conducted in the region. However, it has some limitations. The retrospective nature of the study can lead to recall bias. All patients or their relatives were personally contacted, and hence, mortality was underestimated. Additionally, some factors such as different strains of COVID-19 and different types of vaccination received by patients contributed to disease severity.

## Data Availability

The data that support the findings of this study are available on request from the corresponding author.
